# Case report: Cerebral artery air embolism during CT-guided lung nodule resection in hybrid theater

**DOI:** 10.3389/fsurg.2022.950159

**Published:** 2022-09-26

**Authors:** Huajie Xing, Zhiqiang Wang, Yuequan Jiang

**Affiliations:** Department of Thoracic Oncology, Cancer Hospital, Chongqing University, Chongqing, China

**Keywords:** air embolism, CT, lung nodule, resection, hybrid theater, guided

## Abstract

A middle-aged male received CT-guided lung nodule localization and segmentectomy for a 10-mm lesion in the right upper lung. He developed left side paralysis after surgery, which was proved to be cerebral artery air embolism caused by a CT-guided lung puncture. He achieved almost full recovery with hyperbaric oxygen therapy and intensive rehabilitation. This case highlights the possibility of cerebral artery air embolism during CT-guided lung nodule resection in hybrid theater, with emphasis on prevention and early detection of this life-threatening complication.

## Introduction

Sublobar lung resection has been widely performed for ground-glass opacity dominant lung cancer that requires surgical intervention, and is associated with excellent prognosis. Lung nodule localization is required for lesions that are unable to palpate during minimally invasive surgery. In recent years, intraoperative computed tomography (CT) guided lung nodule localization in hybrid theater has gained increasing popularity, with the advantages of less pain, less radiation exposure, and fewer complications compared to traditional preoperative nodule localization (1). Air embolism is a very rare complication after CT-guided lung nodule localization, but it could cause severe consequences. Here we present a rare case of cerebral artery air embolism after CT-guided lung nodule localization and resection in hybrid theater.

## Case presentation

**Figure 1:**
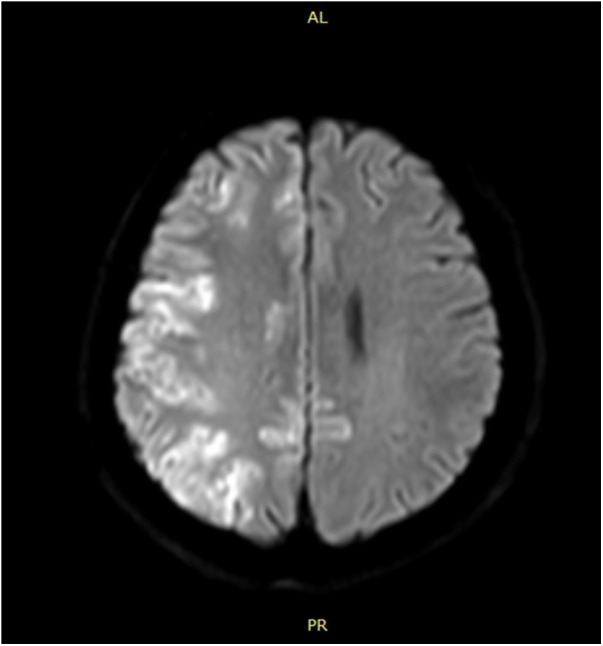
Brain MRI shows widespread cerebral infarction.

A 49-year-old man was referred to our department for a 10-mm part-solid nodule in the posterior segment of the right upper lung. His past history was not significant except for hyperlipaemia. Surgical resection was scheduled. The procedure was performed under general anesthesia. After intubation, the patient was placed in a lateral decubitus position. Then a hook wire was inserted near the nodule under CT guidanc. Uniportal video-assisted thoracic surgery (VATS) segmentectomy was immediately performed in the hybrid theater. The operation took approximately 80 min in total. When the patient woke up from anesthesia, he was found to have paralysis in the left extremities. His left Babinski sign was positive. A brain CT scan was immediately performed, which was normal. These neurological symptoms suggested acute brain infarction and cerebral thromboembolism was highly suspected. So, 2 h later urgent digital subtraction angiography (DSA) was performed in an attempt to remove the thrombus. However, no thrombus was found in this procedure. Then, 10 h later magnetic resonance imaging of the brain showed widespread infarctions in the right occipital lobe and frontoparietal cortices (Figure 1).

**Figure 2:**
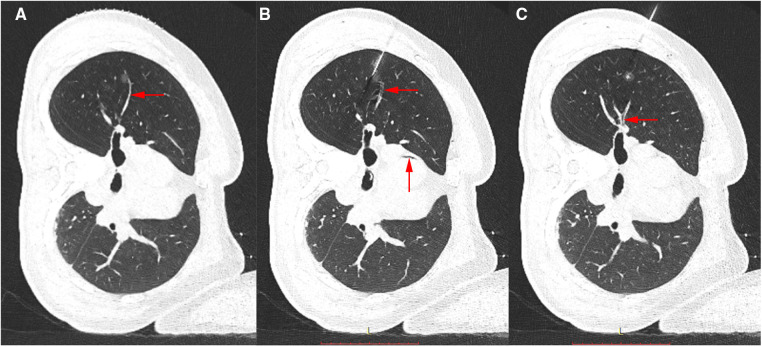
(**A**) CT image of the lung nodule before hook wire insertion. Arrow shows a branch of V2b. (**B,C**) CT image after localization. Arrows show a filling defect in the branch of V2b and the ascending aorta, which is in accordance with air embolism.

Brain infarction was confirmed, but thromboembolism was excluded. The patient had no risk factor for fat embolism such as bone fracture. Thus, air embolism was highly suspected. Then a careful review of the CT scan after hook wire placement surprisingly revealed small air blebs in the ascending aorta and the branch of V2b, which indicated cerebral artery air embolism (Figure 2). The patient received hyperbaric oxygen therapy for 10 days and intensive rehabilitation with almost full recovery, except for mild left-sided weakness.

## Discussion

Systemic air embolism is a very rare complication after CT-guided lung puncture, with a reported incidence at 0.001%–0.003% (2), but it could cause severe consequences including cardiac and brain infarction. Studies on this topic are mostly case reports and focus on CT-guided lung biopsy and radiofrequency ablation. To the best of our knowledge, this is the first report of cerebral artery air embolism after CT-guided lung nodule localization and resection in hybrid theater.

The possible mechanism responsible for air embolism is the formation of a broncho-vascular fistula. As pulmonary vein pressure is low, it is easy to suppose that air can enter the circulation through the fistula in case of accidental pulmonary vein injury. In this case, several risk factors existed. A branch of V2b is near the nodule and the needle tip could have injured the vein in the puncture pathway. The patient was placed in a lateral decubitus position, which led to lower vein pressure since the needle tip was located above the left atrium. What's more, the ventilator caused a pressure gradient between airspace and vein and could drive air into the vessel.

Detection of air in the circulation by CT scan is direct evidence for the diagnosis of air embolism. However, CT may not always reveal the presence of air in the brain. As in this case, air may be absorbed quickly if there is a delay in imaging. Rika Yoshida *et al* reported the time-dependent changes in systemic air embolism during CT-guided lung puncture (3). The interval time in which the air disappears may depend on the amount, which could be as short as 15 min. Thereby the diagnosis of air embolism sometimes requires to be made by exclusion. In this case, early diagnosis of cerebral air embolism is more challenging since the patient was under general anesthesia. Thus, a careful review of the whole-lung CT scan after insertion of hook wire and keeping an eye on any abnormal air in the circulation is crucial, which was ignored in this case.

Once air is detected in circulation, it's important to prevent air blebs from moving into the cerebral arteries, and the Trendelenburg position is suggested. In a care report by Cristian Rapicetta, the air bubble in the pulmonary vein migrated into the descending aorta after adopting the Trendelenburg position, causing no neurological deterioration (4). Once stabilized, hyperbaric oxygen is the most beneficial treatment (5). Hyperbaric oxygen therapy should be started as early as possible. Patients treated with hyperbaric oxygen within 5–7 h from symptom onset have better outcomes (6).

In conclusion, this case illustrates that cerebral artery air embolism should be considered in patients with neurological deterioration after CT-guided lung nodule localization and resection in hybrid theater. Holding the ventilator and avoiding crossing small vessels during puncture could lower the risk of air embolism. A routine CT scan is amenable after insertion of the hook wire for the detection of potential air in the circulation. If air is detected, the Trendelenburg position is recommended, and hyperbaric oxygen treatment is beneficial for neural rehabilitation.

## Data Availability

The original contributions presented in the study are included in the article/Supplementary Material, further inquiries can be directed to the corresponding author/s.
